# Supplementary material for: Vibrio cholerae senses human enteric α-defensin 5 through a CarSR two-component system to promote bacterial pathogenicity
Source: Commun Biol. 2022 Jun 8;5:559. doi: 10.1038/s42003-022-03525-3 (PMC9178039; doi:10.1038/s42003-022-03525-3)
Supplement: Supplementary file 2 — Supplementary Information [file 42003_2022_3525_MOESM2_ESM.pdf]

SUPPLEMENTARY INFORMATION

Vibrio cholerae senses human enteric α-defensin 5 through a CarSR two-component system to promote bacterial pathogenicity

Yutao Liu[1, #], Tingting Xu[2, #], Qian Wang[1], Junxi Huang[2], Yangfei Zhu[3], Xingmei Liu[1], Ruiying Liu [1], Bin Yang[1, *], Kai Zhou[2, *]

[1] TEDA Institute of Biological Sciences and Biotechnology, Nankai University, TEDA, Tianjin 300457, P. R. China;

[2] Shenzhen Institute of Respiratory Diseases, Shenzhen People's Hospital (The Second Clinical Medical College, Jinan University; The First Affiliated Hospital, Southern University of Science and Technology), Shenzhen 518020, Guangdong, P. R. China;

[3]Laboratory Department of Shenzhen People's Hospital (The Second Clinical Medical College, Jinan University; The First Affiliated Hospital, Southern University of Science and Technology), Shenzhen 518020, Guangdong, P. R. China

[#] Yutao Liu, Tingting Xu contributed equally to this work.

[*] To whom correspondence may be addressed. E-mail: yangbin@nankai.edu.cn or Kai_Zhou@zju.edu.cn

This Supplementary Information includes:

Supplementary Figures 1 to 4

Supplementary Tables 1 to 2

a

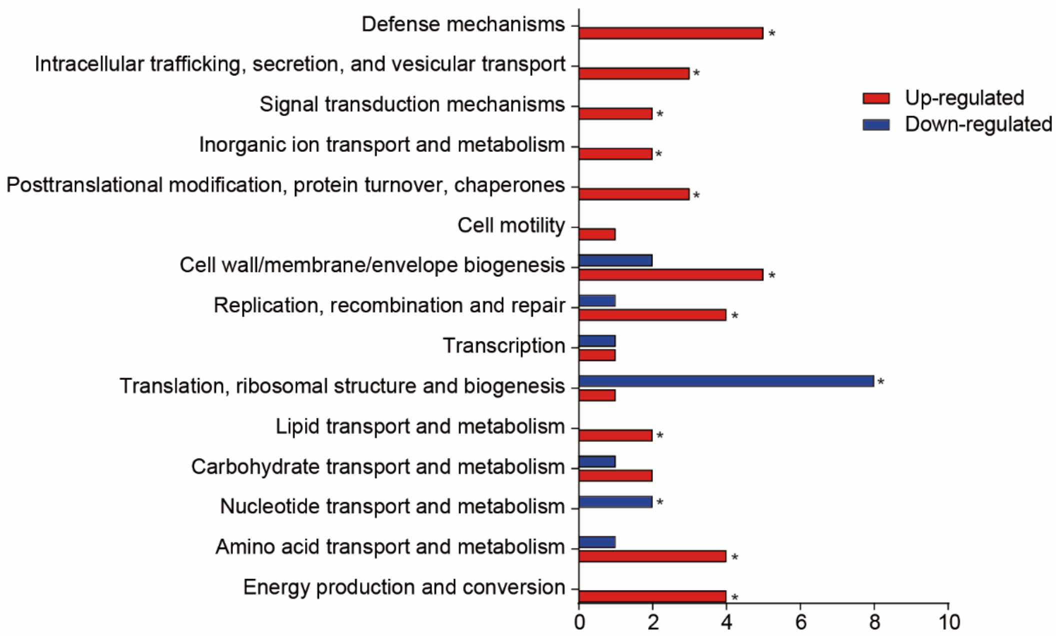

Supplementary Figure 1. (**a**) COG analysis of HD-5-induced genes in *V. cholerae*. Bars represent the number of upregulated (red) or downregulated (blue) genes. The significant enrichment of a given COG in the sets of up- or downregulated genes was determined using Fisher's exact test. COG categories that are significantly enriched are indicated by asterisks (*P*<0.05).

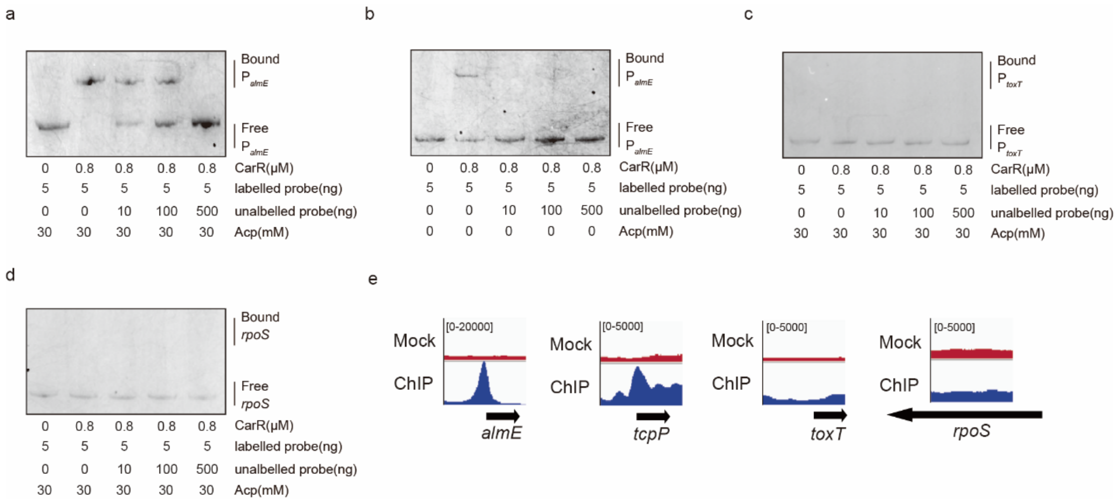

Supplementary Figure 2. (**a, b**) CarR bound to P*almE* with (**a**) or without (**b**) 30mM acetyl phosphate. (**c, d**) CarR bound to P*toxT* and *rpoS* with 30mM acetyl phosphate (negative control). (**e**) Original sequence peaks of the CarR binding regions in the P*almE*, P*tcpP*, P*tpxT*, *rpoS* according to ChIP-seq analyses.

a

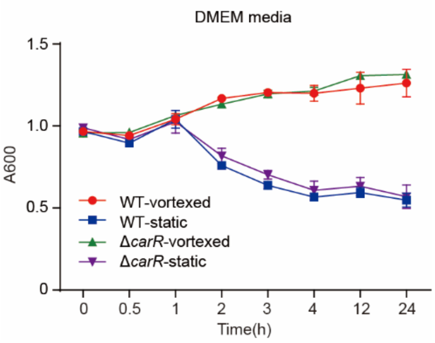

Supplementary Figure 3. (**a**) Autoaggregation assays of CarR. Data are presented as mean ± SD (n = 3).

a

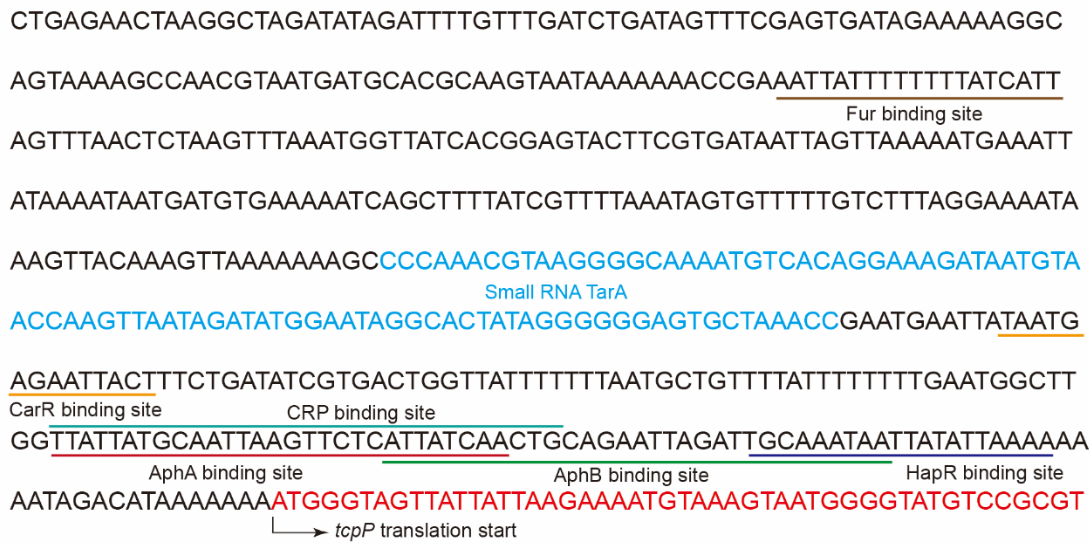

Supplementary Figure 4. (**a**) Promoter organization of *tcpP*. Sequences were derived from *V. cholerae* EL2382. The translation start site of *tcpP* is indicated by bent arrows. The binding sites of different regulators in the promoter region of *tcpP* are underlined with solid lines. Small RNA TarA is outlined in blue.

Supplementary Tables

Supplementary Table 1. Strains and plasmids used in this study.

**Strains**	**Genotype or description**	**Source**
E12382	*Vibrio Cholerae* O1 El Tor strain E12382	Shanghai CDC*
S-17	*E. coli* S17-1/λpir strain	Lab collection
DH5α	*E. coli* DH5α/λpir strain	Lab collection
Δ*carR*	*carR* deletion mutant in E12382	This work
Δ*tcpP*	*tcpP* deletion mutant in E12382	This work
Δ*carR*Δ*tcpP*	*tcpP* deletion mutant in Δ*carR*	This work
Δ*carR*Δ*lacZ*	*carR* deletion mutant in Δ*lacZ*	This work
Δ*ctxAB*	*ctxAB* deletion mutant in E12382	This work
WT (pBAD33)	E12382 containing pBAD33	This work
Δ*lacZ*(pBAD33)	Δ*lacZ* containing pBAD33	This work
Δ*carR*+	Δ*carR* containing pBAD33-*carR*	This work
RstA-ChIP	Δ*carR* containing pBAD33-*carR*-3×FLAG	This work
GV26	BL21(DE3) containing pET28a-*carR*	This work
**Plasmids**		
pBAD33	Bacteria expression vector, CmR	Lab collection
pRE112	Suicide vector for mutation, CmR	Lab collection
pET28a	T7 expression vector, KmR	Lab collection
pBAD33-*carR*	pBAD33 carrying *rstA* from E12382，CmR	This work
pBAD33-*carR*-FLAG	pBAD33 carrying *rstA*-3x FLAG from E12382，CmR	This work

*, Shanghai CDC, Shanghai Municipal Center for Disease Control & Prevention, China.

Supplementary Table 2. Primers used in this study (5'-3').

Primers for gene mutation		
carR	P1	GCTCTAGACATCCAAATCGAGCGCTTTACCGA
carR	P2	GGTTTCCTTTAAATCAACTTAGGTGGGGACCTCGTATTTACGGTTTAT
carR	P3	ATAAACCGTAAATACGAGGTCCCCACCTAAGTTGATTTAAAGGAAAC
carR	P4	CGGAGCTCTTACGGATGAGGTTAACCAATGCC
tcpP	P1	GGGGTACCAAAAGCCAACGTAATGATGCACGC
tcpP	P2	GACCTGTAGCGCCCCCCCAAGCTTTATTTACATTTTCTTAATAATAA
tcpP	P3	TTATTATTAAGAAAATGTAAAGTAAAGCTTGGGGGGGCGCTACAGGTC
tcpP	P4	TCCCCCGGGCAACCGCGAACATTAGGGTAAAGA
lacZ	P1	CGGGATCCAGACCGGGAGCTAACGTA
lacZ	P2	GGCTTATTGTGGATCGGAGAAGTTGCGCATAAG
lacZ	P3	CCACAATAAGCCAGAGAGCCT
lacZ	P4	GTCTCGAGGATCGCTGTATTTCACTCG
ctxAB	P1	GGGGTACCCTACCACAGCGGACAGGCTCTA
ctxAB	P2	TTAAACAAAGGGAGCATTATGATATAAAAAAGCCCACCTC
ctxAB	P3	GAGGTGGGCTTTTTTATATCATAATGCTCCCTTTGTTTAA
ctxAB	P4	TCCCCCGGGCAGTCAAAGGCTACTGTTGGGAA
Primers for pBAD33 identifying		
pBAD33	F	ATGCCATAGCATTTTTATCC
pBAD33	R	GATTTAATCTGTATCAGG
Primers for complement construction		
carR	F	CGAGCTCATGTCTAACCAACCCAGTCTCT
carR	R	CTCTAGATTACCAAGTGTCAGGCACAAATA
Primers for ChIP strain construction		
carR	F	CGGAGCTCATGTCTAACCAACCCAGTCTCT
carR	R	CTCTAGATTACTATTTATCGTCGTCATCTTTGTAGTCGATATCATGAT<br>CTTTATAATCACCGTCATGGTCTTTGTAGTCCCAAGTGTCAGGCACAA
Primers for protein purification		
carR	F	CATGCCATGGCCATGTCTAACCAACCCAGTCTCT
carR	R	GGCTCGAGCCAAGTGTCAGGCACAA
Primers for DNase I foot-printing		
P_{tcpP}	F	TGGAATAGGCACTATAGGGG
P_{tcpP}	R	GGGAAATTGATAAATCACGC
Primers for EMSA		
P_{almE}	F	GATGTTGCGTCTATTGGC
P_{almE}	R	CTGTTTAACCCATAATGCAG
P_{tcpP}	F	TGGAATAGGCACTATAGGGG
P_{tcpP}	R	GGGAAATTGATAAATCACGC
P_{toxT}	F	AAATTCTAATTATAAAACGCAAAT
P_{toxT}	R	TGCGTTCTACTCTGAAGATATATA

rpoS	F	CGTGCGGTTGAGAAATTCGA
rpoS	R	TGCCTTATCTCCATCCCCAC
Primers for qRT-PCR		
rrsA	F	ACCTTACCTACTCTTGACATCCA
rrsA	R	CCCAACATTTCACAACACGAG
carR	F	AACCAAAGCCGACGCCACTG
carR	R	CCATCATACTCAATCCCCCGAAG
carS	F	GCGAGCATTATTGAGGACACGGA
carS	R	AAGGGTTGCAGAGAAGGGAGGT
tcpP	F	ATGGGGTATGTCCGCGTGAT
tcpP	R	TTTGGACAGGGGGCAGGATG
toxT	F	CGTTGGGCAGATATTTGTGGTG
toxT	R	CACTTGGTGCTACATTCATGGTTG
tcpA	F	CATTCGCAATTACAGTCGGTGG
tcpA	R	CAAAGCTTCTCAACATGCGTGA
ctxA	F	CTCAGACGGGATTTGTTAGGC
ctxA	R	CTATCTCTGTAGCCCCTATTACG
almE	F	AACAGATCCAAGAGACGCTGA
almE	R	GACGTAACGGTTCCACATCC
Primers for ChIP-qRT		
P_{vc1318}	F	TGCCAGTAAACAAGCAGTGA
P_{vc1318}	R	CGAATGTAAGTTTGGGCAGC
$P_{vca1078}$	F	CGCGAGCTGTAATGTTGACT
$P_{vca1078}$	R	TGCGGCGATATGATACCAAG
P_{vc0973}	F	AGTTGGTTAGATTTGCCCTGG
P_{vc0973}	R	GCACCCAGAAGAGTACGTTT
P_{vc0633}	F	ATTAGATTGCGTGCATTTG
P_{vc0633}	R	AGCAATCAGAGTCTTGTTCATCT
$P_{vca0227}$	F	GGCTCAATGGGACTGGAAAC
$P_{vca0227}$	R	GCAGCCCAATGAATACGAGA
P_{almE}	F	TGAACAGTTAGGGTTAGCGGT
P_{almE}	R	ATGGCATTGTCAGCTACCGA
P_{tcpP}	F	GGGGCAAAATGTCACAGGAA
P_{tcpP}	R	TGATAAATCACGCGGACATACC
P_{toxT}	F	CAAGTGGTCAAATACTATGTTCTCA
P_{toxT}	R	TCCCAATCATTGCGTTCTACTC
rpoS	F	CGTGCGGTTGAGAAATTCGA
rpoS	R	TGCCTTATCTCCATCCCCAC
