# Supplementary material for: Vibrio cholerae senses human enteric α-defensin 5 through a CarSR two-component system to promote bacterial pathogenicity
Source: Commun Biol. 2022 Jun 8;5:559. doi: 10.1038/s42003-022-03525-3 (PMC9178039; doi:10.1038/s42003-022-03525-3)
Supplement: Supplementary file 3 — Description of Additional Supplementary Files [file 42003_2022_3525_MOESM3_ESM.pdf]

Description of Additional Supplementary Files

File name: Supplementary Data 1
Description: RNA-seq analysis of V. cholerae grown in presence of HD-5.

File name: Supplementary Data 2
Description: ChIP-seq analysis of CarR potential targets in V. cholera.

File name: Supplementary Data 3
Description: The source data behind the graphs in the paper.
