# Supplementary material for: Vibrio cholerae senses human enteric α-defensin 5 through a CarSR two-component system to promote bacterial pathogenicity
Source: Commun Biol. 2022 Jun 8;5:559. doi: 10.1038/s42003-022-03525-3 (PMC9178039; doi:10.1038/s42003-022-03525-3)
Supplement: Supplementary file 7 — Reporting Summary [file 42003_2022_3525_MOESM7_ESM.pdf]

nature portfolio

Corresponding author(s): Kai Zhou

Last updated by author(s): Apr 29, 2022

Reporting Summary

Nature Portfolio wishes to improve the reproducibility of the work that we publish. This form provides structure for consistency and transparency in reporting. For further information on Nature Portfolio policies, see our Editorial Policies and the Editorial Policy Checklist.

Statistics

For all statistical analyses, confirm that the following items are present in the figure legend, table legend, main text, or Methods section.

n/a	Confirmed	
☐	☒	The exact sample size (*n*) for each experimental group/condition, given as a discrete number and unit of measurement
☐	☒	A statement on whether measurements were taken from distinct samples or whether the same sample was measured repeatedly
☐	☒	The statistical test(s) used AND whether they are one- or two-sided<br>*Only common tests should be described solely by name; describe more complex techniques in the Methods section.*
☒	☐	A description of all covariates tested
☐	☒	A description of any assumptions or corrections, such as tests of normality and adjustment for multiple comparisons
☐	☒	A full description of the statistical parameters including central tendency (e.g. means) or other basic estimates (e.g. regression coefficient) AND variation (e.g. standard deviation) or associated estimates of uncertainty (e.g. confidence intervals)
☐	☒	For null hypothesis testing, the test statistic (e.g. *F*, *t*, *r*) with confidence intervals, effect sizes, degrees of freedom and *P* value noted<br>*Give P values as exact values whenever suitable.*
☒	☐	For Bayesian analysis, information on the choice of priors and Markov chain Monte Carlo settings
☐	☒	For hierarchical and complex designs, identification of the appropriate level for tests and full reporting of outcomes
☒	☐	Estimates of effect sizes (e.g. Cohen's *d*, Pearson's *r*), indicating how they were calculated

Our web collection on statistics for biologists contains articles on many of the points above.

Software and code

Policy information about availability of computer code

Data collection	Sequencing data was collected by Illumina HiSeq 2500 (Illumina).
Data analysis	MedCalc (v12.3.0.0); Microsoft Excel 2019 (16.0.14026.20294); GraphPad Prism(v7.0.4); ImageJ (v1.8.0); DESeq2; Integrative Genomics Viewer(2.11.3); BWA mem (version 0.7.12); Cutadapt (v1.9.1); MACS2 (v2.1.0); Peak Scanner (v1.0)

For manuscripts utilizing custom algorithms or software that are central to the research but not yet described in published literature, software must be made available to editors and reviewers. We strongly encourage code deposition in a community repository (e.g. GitHub). See the Nature Portfolio guidelines for submitting code & software for further information.

Data

Policy information about availability of data

All manuscripts must include a data availability statement. This statement should provide the following information, where applicable:

- Accession codes, unique identifiers, or web links for publicly available datasets
- A description of any restrictions on data availability
- For clinical datasets or third party data, please ensure that the statement adheres to our policy

The RNA-seq data have been deposited in the NCBI Sequence Read Archive database under accession code SRR16991885 (HD-5), SRR16991886 (HD-5), SRR16991887 (HD-5), SRR16991888 (CT), SRR16991889 (CT) and SRR16991890 (CT).
The ChIP-seq data have been deposited in the NCBI Sequence Read Archive database under accession code SRR17012211(ChIP-mock) and SRR17012212(ChIP-RstA).

Field-specific reporting

Please select the one below that is the best fit for your research. If you are not sure, read the appropriate sections before making your selection.

☒ Life sciences ☐ Behavioural & social sciences ☐ Ecological, evolutionary & environmental sciences

For a reference copy of the document with all sections, see nature.com/documents/nr-reporting-summary-flat.pdf

Life sciences study design

All studies must disclose on these points even when the disclosure is negative.

Sample size	RNA-seq was performed three times for the CT and HD-5 sample. The RNA collected from three different biological samples of the CT and HD-5 were used for analysis. All other in vitro experiments were repeated at least three times ($n \geq 3$). Mice colonization experiments were conducted twice with at least 3 mice ($n \geq 3$) in each group, and the combined data for the two experiments was used for statistical analysis.
Data exclusions	No data were excluded from the analyses.
Replication	All the reported experiments were reproducible. Data reproducibility was confirmed by three independent experiments. RNA-seq results were validated by three independent qRT-PCR analyses on target genes.ChIP-seq results were validated by three independent ChIP-qPCR analyses on target peaks.
Randomization	All samples were assigned to groups randomly.
Blinding	cDNA and ChIP-seq libraries were constructed and analyzed by NOVOGENE, Inc (TianJin, China), which also provided the statistical analysis. Other experiments were not done blindly. Most experiments were conducted by at least two different researchers who have not known the situation and results of the study in advance and repeated on at least two independent days.

Reporting for specific materials, systems and methods

We require information from authors about some types of materials, experimental systems and methods used in many studies. Here, indicate whether each material, system or method listed is relevant to your study. If you are not sure if a list item applies to your research, read the appropriate section before selecting a response.

Materials & experimental systems

n/a	Involved in the study	
☐	☒	Antibodies
☐	☒	Eukaryotic cell lines
☒	☐	Palaeontology and archaeology
☐	☒	Animals and other organisms
☒	☐	Human research participants
☒	☐	Clinical data
☒	☐	Dual use research of concern

Methods

n/a	Involved in the study	
☐	☒	ChIP-seq
☒	☐	Flow cytometry
☒	☐	MRI-based neuroimaging

Antibodies

Antibodies used	Anti-CarR monoclonal antibody (custom-made by Willget Biotech Co., Ltd). 1:2,000 dilution used for immunoblotting.<br>Anti-RNA polymerase beta antibody cat.ab191598. Abcam. 1:2,000 dilution used for immunoblotting.<br>Anti-cholera toxin antibody cat.ab123129. Abcam. 1:2,000 dilution used for immunoblotting.<br>HRP-conjugated goat anti-rabbit IgG secondary antibody cat.EF0002. Sparkjade. 1:5,000 dilution used for immunoblotting.
Validation	Anti-RNA polymerase beta antibody : rabbit monoclonal to RNA polymerase beta; Suitable for: IP, WB; Reacts with: Escherichia coli.<br>Anti-cholera toxin antibody: Rabbit polyclonal to Cholera Toxin; Suitable for: WB, ELISA; Reacts with: Other species.

Eukaryotic cell lines

Policy information about cell lines

Cell line source(s)	Caco-2 cell line were purchased from the Shanghai<br>Institute of Biochemistry and Cell Biology of the Chinese Academy of Sciences (Shanghai, China).
Authentication	All the cell lines of the Shanghai Institute of Biochemistry and Cell Biology are originated from ATCC. ATCC authenticates its

Authentication	cell lines through morphology, karyotyping, and STR analyses, thus Caco-2 cells were not authenticated after receipt. Regular inspection of cell culture for coherent morphology with ATCC source images was routinely performed.
Mycoplasma contamination	Cells routinely tested negative for mycoplasma contamination.
Commonly misidentified lines (See ICLAC register)	No commonly misidentified cell lines were used.

Animals and other organisms

Policy information about studies involving animals; ARRIVE guidelines recommended for reporting animal research

Laboratory animals	Both sexes of CD-1 infant mice (5 days old)
Wild animals	This study did not involve wild animals.
Field-collected samples	This study did not involve samples collected from the field.
Ethics oversight	All animal studies were conducted according to protocols approved by the Institutional Animal Care Committee of Nankai University (Tianjin, China) and performed under protocol no. IACUC 2016030502.

Note that full information on the approval of the study protocol must also be provided in the manuscript.

ChIP-seq

Data deposition

☒ Confirm that both raw and final processed data have been deposited in a public database such as GEO.

☒ Confirm that you have deposited or provided access to graph files (e.g. BED files) for the called peaks.

Data access links<br>*May remain private before publication.*	https://dataview.ncbi.nlm.nih.gov/object/PRJNA782689
Files in database submission	Input_2_1.fq.gz(mock-ChIP); Input_2_2.fq.gz(mock-ChIP); Output_2_1.fq.gz(RstA-ChIP); Output_2_2.fq.gz(RstA-ChIP)
Genome browser session (e.g. UCSC)	https://www.ncbi.nlm.nih.gov/assembly/GCF_007624355.1

Methodology

Replicates	ChIP-seq was performed once for the ChIP sample (RstA-ChIP) and the mock ChIP sample (mock-ChIP). The promoter region of tcpP and almE were significantly enriched in the ChIP sample compared to that in the mock ChIP sample. The ChIP-qPCR results (from three independent biological experiments) confirmed the enrichment of the tcpP and almE promoter region in the ChIP sample.
Sequencing depth	Sequencing layout: 2×150 paired-end<br>Sequencing Depth for each sample (ID: total number of reads/uniquely mapped)<br>Input_2_1.fq.gz:15299984/15293186<br>Input_2_2.fq.gz:15710719/15703859<br>Output_2_1.fq.gz:22092320/22077873<br>Output_2_2.fq.gz:12512037/12504163
Antibodies	Anti-FLAG mouse monoclonal antibody (Sigma #F1804)
Peak calling parameters	Peaks were called using MACS2 with default settings and the fragment size set to 150 bp (option '--extsize 150').
Data quality	Only uniquely mapped reads with an alignment score ≥ 20 were used for peak calling. After mapping reads to the reference genome, the MACS2 (v2.1.0) peak finding algorithm was used to identify regions of IP enrichment over control. A q value threshold of enrichment of 0.05 was used for all data sets.
Software	Quality control: Cutadapt (v1.9.1)<br>Read mapping: BWA mem (v0.7.12)<br>Peak calling: MACS2 (v2.1.0)<br>Visual analysis:Integrative Genomics Viewer(2.11.3)
